# 

**Published:** 1889-02

**Authors:** 


CONTENTS.

VOL. 36 No. 2.

| | PAGE. |
|---|---|
| Special | 25 |
| Index | 25 |
| Buddhism and Theosophy | 25 |
| Hygienic Heresy | 26 |
| The Human Heart | 28 |
| The Progress of Hindooism in the United States | 29 |
| The Chemistry of Breadmaking | 31 |
| A Remarkable Sensitive | 33 |
| Amateur Photography | 35 |
| The Future Life | 36 |
| A Walking Encyclopedia | 37 |
| A Paralytic's Recovery | 38 |
| Influence of Music upon Animals | 38 |
| Curious Cradles | 40 |
| Was it Mind-Reading? | 41 |
| The Prayer of a Blind Woman | 43 |
| Book Notices | 44 |
| Household | 45 |
| Miscellaneous | 47 |
| Procrastination | 48 |

INDEX TO ADVERTISEMENTS OF HALF A PAGE AND OVER.

| | PAGE. |
|---|---|
| Murdock's Liquid Food Co | I |
| The Ozone Disinfector and Deoderizer | II |
| The Trained Nurse | II |
| The Banner of Life and Home Physician | III |
| Hollow Supposit ries | IV |
| Lactopeptine | V |
| The Family Cyclopædia of Useful Knowledge | VI |
| Revised Premium List | VIII |
| Rio Chemical Co | X |
| The Her› Cure Co | XI |

